# Dementia in people with severe or profound intellectual (and multiple) disabilities: Focus group research into relevance, symptoms and training needs

**DOI:** 10.1111/jar.12912

**Published:** 2021-07-02

**Authors:** Alain D. Dekker, Maureen B. G. Wissing, Aurora M. Ulgiati, Bas Bijl, Gaby van Gool, Marieke R. Groen, Esther S. Grootendorst, Ina A. van der Wal, Johannes S. M. Hobbelen, Peter P. De Deyn, Aly Waninge

**Affiliations:** ^1^ Department of Neurology and Alzheimer Center University of Groningen, University Medical Center Groningen Groningen The Netherlands; ^2^ Department of Practice‐Oriented Scientific Research (PWO) Alliade Care Group Heerenveen The Netherlands; ^3^ Research Group Healthy Ageing, Allied Health Care and Nursing Hanze University of Applied Sciences Groningen The Netherlands; ^4^ Advisium 's Heeren Loo Amersfoort The Netherlands; ^5^ Ipse de Bruggen Zoetermeer The Netherlands; ^6^ Department of General Practice & Elderly Care Medicine University of Groningen, University Medical Center Groningen Groningen The Netherlands; ^7^ Institute Born‐Bunge University of Antwerpen Antwerp Belgium; ^8^ Department of Neurology and Memory Clinic Hospital Network Antwerp (ZNA) Middelheim and Hoge Beuken Antwerp Belgium; ^9^ Department of Health Psychology University of Groningen, University Medical Center Groningen Groningen The Netherlands; ^10^ Royal Dutch Visio Vries The Netherlands

**Keywords:** dementia, Down syndrome, focus groups, intellectual disabilities, severe or profound intellectual (and multiple) disabilities

## Abstract

**Background:**

Differentiating dementia from baseline level of functioning is difficult among people with severe/profound intellectual (and multiple) disabilities. Moreover, studies on observable dementia symptoms are scarce. This study examined (a) the relevance of dementia diagnosis, (b) observable symptoms and (c) training/information needs.

**Methods:**

Four explorative focus groups were held with care professionals and family members who have experience with people with severe/profound intellectual (and multiple) disabilities (≥40 years) and decline/dementia.

**Results:**

Thematic analysis showed that participants wanted to know about a dementia diagnosis for a better understanding and to be able to make informed choices (question 1). Using a categorisation matrix, cognitive and behavioural changes were shown to be most prominent (question 2). Participants indicated that they needed enhanced training, more knowledge development and translation, and supportive organisational choices/policies (question 3).

**Conclusions:**

Timely identifying/diagnosing dementia allows for a timely response to changing needs. This requires a better understanding of symptoms.

## 1 INTRODUCTION

In the last decades, life expectancy of people with intellectual disabilities has increased even faster than in the general population (Bittles & Glasson, 2004; Coppus, 2013; Evans et al., 2013). Because advanced age is the greatest risk factor for dementia (Alzheimer's Association, 2020), dementia is a growing challenge in intellectual disability care. People with Down syndrome are at a particularly high genetic risk to develop Alzheimer's disease: approximately 75% would developed dementia by age 65 (Wiseman et al., 2015).

Diagnosing dementia in people with intellectual disabilities is complicated due to the level of intellectual disability, (life‐long) patterns of characteristic/typical behaviour related to the intellectual disability and the presence of comorbidities, which may be associated with dementia‐like symptoms (Dekker et al., 2015; Jamieson‐Craig et al., 2010; Sabbagh & Edgin, 2016; Zigman et al., 2008). Moreover, it may be hard to differentiate between ageing and dementia. The diagnosis of dementia requires the presence of cognitive and behavioural decline from a previous higher level of functioning, and this decline must interfere with daily functioning (American Psychiatric Association, 2013; Fletcher et al., 2016; Sabbagh & Edgin, 2016; Zigman et al., 2008). However, the more severe and complex the present disabilities, the more difficult the assessment of decreasing skills due to dementia. This is particularly difficult in people with severe or profound intellectual (and multiple) disabilities (Evans et al., 2013; McKenzie et al., 2018).

For this population, there are hardly any validated direct neuropsychological tests and informant‐based dementia questionnaires available for (early) identification and diagnosis of dementia (Elliott‐King et al., 2016; Esbensen et al., 2017; Fletcher et al., 2016; Hon et al., 1999; Keller et al., 2016; McKenzie et al., 2018). A diagnosis of dementia in this specific population is currently based on multidisciplinary clinical assessment (by experienced clinicians) involving observations, informant interviews and/or screening case notes (Day, 1985; Duggan et al., 1996; Evenhuis, 1990; Määttä et al., 2006; Margallo‐Lana et al., 2007; Reid & Aungle, 1974; Sauna‐aho et al., 2018). Moreover, studies on dementia symptoms in people with severe/profound intellectual (and multiple) disabilities are scarce because scientific research has primarily focused on dementia in people with mild/moderate intellectual disabilities (Wissing et al., 2021).

People with severe/profound intellectual (and multiple) disabilities have an estimated IQ of less than 35. Besides, they often experience serious health problems and sensory impairments that may adversely affect their functioning (Nakken & Vlaskamp, 2007; van Timmeren et al., 2016). In addition, they often experience profound neuromotor dysfunctions (Nakken & Vlaskamp, 2007). In these persons, it is difficult to differentiate deterioration due to dementia from the severe or profound pre‐existing limitations in functioning. Firstly, it is difficult to assess cognitive decline due to the developmental age below 36 months. Although memory changes are indicative of dementia in people with mild intellectual disabilities, decline in daily functioning is more visible in people with more severe intellectual disabilities (Jamieson‐Craig et al., 2010). However, people with severe/profound intellectual (and multiple) disabilities often need lifelong support. They have never developed specific skills and have to be supported by care professionals for certain tasks. As a result, such skills cannot be considered as symptoms indicative of dementia (Llewellyn, 2011; Sheehan et al., 2015). Secondly, communication is mostly non‐verbal and, therefore, there are no self‐reported symptoms (Nakken & Vlaskamp, 2007; Smiley & Cooper, 2003). Thirdly, currently used dementia questionnaires are not suitable for severe/profound intellectual (and multiple) disabilities, and direct neuropsychological assessments are almost impossible due to floor effects (Elliott‐King et al., 2016; Esbensen et al., 2017; Fletcher et al., 2016; Hon et al., 1999; Keller et al., 2016; McKenzie et al., 2018). Fourthly, it is difficult to assess dementia‐related decline due to the frequent presence of multiple concurrent health problems (van Timmeren et al., 2017).

Another obstacle for early identification and monitoring of deterioration in people with severe/profound intellectual (and multiple) disabilities is the dependence on observations of informants, such as family members and direct support professionals/caregivers (DSPs) (McKenzie et al., 2018), who often lack necessary background knowledge (Cleary & Doody, 2017; Iacono et al., 2014) partly because information about symptoms and course of dementia in this population has been scarce until now (Wissing et al., 2021). On the other hand, family members and care professionals are often able to give concrete examples of minor signs of decline that they have observed. Until now, this knowledge has been individual‐based and linked to one or a few people with severe/profound intellectual (and multiple) disabilities. Therefore, there is a great need for knowledge and education about dementia in this population in daily practice.

An explorative study paves the way for further research on dementia in people with severe/profound intellectual (and multiple) disabilities. This study focused on three practice‐based questions:

- Why is it important to know if an individual with severe/profound intellectual (and multiple) disabilities has dementia? (question 1)
- Which dementia symptoms in people with severe/profound intellectual (and multiple) disabilities are recognised in daily practice? (question 2)
- What are training/information needs regarding dementia in people with severe/profound intellectual (and multiple) disabilities? (question 3)

## 2 METHODS

### 2.1 Study consortium

This focus group study was part of the research project ‘Practice‐based questions about dementia in people with severe or profound intellectual (and multiple) disabilities’, a collaborative effort of Hanze University of Applied Sciences, University of Groningen and University Medical Center Groningen (UMCG) with four Dutch care institutions (Ipse de Bruggen, 's Heeren Loo, Talant (part of Alliade Care Group) and Royal Dutch Visio) throughout The Netherlands, representative for the Dutch situation due to the high number of people with severe/profound intellectual (and multiple) disabilities for whom they provide care and treatments.

### 2.2 Study design

This explorative study was based on a qualitative research method using focus groups. Focus groups are group interviews that are not aimed at immediate problem‐solving but at identifying practice‐based experiences, attitudes and needs regarding a particular problem. Interaction between participants is key (Van Royen & Peremans, 2007). We held four explorative focus group sessions with 11–13 participants each. To conduct and report this focus group study, we largely followed the method described by Breen (2006), the Consolidated Criteria for Reporting Qualitative Research (COREQ) (Tong et al., 2018) and Van Royen and Peremans (2007).

### 2.3 Participants

Participants were selected based on the criterion that they would have something to say about dementia in people with severe/profound intellectual disabilities, that is, purposive sampling (Rabiee, 2004). Participants were purposefully selected using a two‐stage procedure. First of all, care professionals and family members with experience with people (≥40 years) with severe/profound intellectual disabilities (established according to dossier and clinical judgement) and showing decline/dementia (with/without Down syndrome; with/without another (e.g., visual or motor) disability) were identified through contact persons at the four care institutions, the research advisory board in which family members and care professionals participated and through the project team members' network (snowball sampling method). In this process, the professions of potential participants were considered to ensure that the focus groups were multidisciplinary (like in daily practice). Therefore, the number of physicians/nurse specialists, allied health care professionals (occupational therapists, physiotherapists and speech therapists), psychologists (behavioural therapists who studied psychology or special needs education), psychological assistants, DSPs and family members were noted. If necessary, additional people were identified. A total of 53 potential participants were identified. This purposive sample subsequently received an invitation to participate by e‐mail. Four people could not attend, resulting in 49 participants for this focus group study. These 49 participants were divided into the four focus groups in a multidisciplinary manner, that is, based on care professional versus family member and based on different professions.

### 2.4 Ethics and consent

The Medical Ethical Committee of the UMCG decided that the Dutch Medical Research Human Subjects Act did not apply to this study (METc 2019/198). The study was registered in the UMCG Research Register (no. 201900193) and conducted in accordance with the UMCG Research Code and the EU General Data Protection Regulation. Each participant gave written consent for audiotaping of the focus group and analysis of this combined with questionnaire data.

### 2.5 Data collection

#### 2.5.1 General participants' characteristics

Participants filled in a questionnaire stating their age, sex, highest level of education and relationship to people with severe/profound intellectual (and multiple) disabilities. Care professionals also stated how many years they have worked with people with severe/profound intellectual (and multiple) disabilities in general. Moreover, they answered on how frequently they work with people with severe/profound intellectual (and multiple) disabilities as well as with those with severe/profound intellectual (and multiple) disabilities and decline/dementia, respectively.

#### 2.5.2 Focus group procedure

Four simultaneous focus group sessions were held, each lasting approximately 2 hours with a 15‐min break. Each focus group was led by a moderator with considerable professional experience in intellectual disability care. For reasons of uniformity, the moderators received the same instructions and followed a procedural protocol drawn up in advance (Breen, 2006). Prior to the session, they welcomed participants, checked if participants signed informed consent forms and introduced the topic, the procedure, the rules of play (i.e., focus groups are not aimed at immediate problem‐solving but at exploring and identifying experiences, attitudes and needs), the confidentiality and the multidisciplinary group composition. Furthermore, the three research questions were asked in the aforementioned order. The focus group interviews were semi‐structured with three open research questions to guide the discussion. Moderators monitored time and ensured that all participants were able to speak.

#### 2.5.3 Recording and transcription

The sessions were recorded with Tascam DR‐40V2 digital audio recorders with an external omnidirectional microphone. Audio tapes were transcribed in Dutch (clean transcription) by the University Translation and Correction Service of the University of Groningen Language Centre. Fillers, hesitations and slips of the tongue were left out.

### 2.6 Data analysis

Three authors independently analysed the transcripts using a qualitative method of content analysis called inductive content analysis (Elo & Kyngäs, 2008) also known as thematic analysis (Braun & Clarke, 2006) for question 1 and question 3. Following Braun and Clarke (2006), this analysis consists of five steps. In step 1 (‘familiarising with the data’), the three researchers independently read the full transcripts. In step 2 (‘generating initial codes’), the transcripts were openly coded also independent of each other. In step 3 (‘searching for themes’), the three researchers independently interpreted and divided into categories, which were then divided into overarching (sub)themes. This was an iterative process of reading, categorising, rereading, refining and so forth. In step 4 (‘reviewing themes’), the researchers met, discussed, compared and refined the division into categories and (sub)themes until they had reached consensus. In step 5 (‘defining and naming themes’), phrasing of categories and (sub)themes was tailored to the research question. It is a recursive process, moving back and forth between the steps (Braun & Clarke, 2006). To enhance trustworthiness and clarify participants' opinions and experiences, the thematic description (Section 3) was illustrated with authentic citations (Elo & Kyngäs, 2008). To improve readability, these quotes were linguistically corrected and, where possible, shortened (for instance, by leaving out unnecessary colloquial words) without the original meaning being affected.

For question 2, a qualitative method of content analysis combining aspects of deductive and inductive content analysis was used. Since dementia symptoms in people with severe/profound intellectual (and multiple) disabilities have been hardly studied in literature (Wissing et al., 2021), this study undertook an exploratory approach to collect symptoms based on experiences in daily practice. To structure the broad range of symptoms, a categorisation matrix (Elo & Kyngäs, 2008) was designed based on the most important clusters of dementia symptoms. The matrix rows were deductively designed in line with dementia diagnostic criteria (American Psychiatric Association, 2013; McKhann et al., 2011; World Health Organization, 2010) showing the following themes: cognitive changes, behavioural changes (categories defined in accordance with the BPSD‐DS evaluation scale [Dekker et al., 2018, 2021]), motor changes and medical comorbidities (Strydom et al., 2010). To improve further interpretation, we categorised symptoms based on the daily contexts in which they are often observed in practice (columns). These daily contexts were inductively analysed and defined based on the participants' descriptions of symptoms. In other words, symptoms in people with severe/profound intellectual (and multiple) disabilities mentioned by participants were coded and categorised in a matrix, which was partially deductively designed (rows consist of cluster of symptoms based on criteria/existing literature) and partially inductively designed (columns consist of daily contexts in which symptoms were seen according to participants).

Finally, the three researchers read the transcripts once more to compare these to the categories and (sub)themes that were ultimately defined per research question. This iterative process of reading, categorising, rereading and refining also involved refining the naming of categories and (sub)themes. Since interaction between participants is key in focus groups (Van Royen & Peremans, 2007) and participants thus respond to each other, this study did not intend to perform additional analyses with subgroups of participants. An integrated analysis was aimed for instead. The original Dutch manuscript with selected quotes was translated to English by the University Translation and Correction Service of the University of Groningen Language Centre.

## 3 RESULTS

To learn more about practice‐based experiences, insights and needs regarding dementia in people with severe/profound intellectual (and multiple) disabilities, four focus group sessions were held with 13, 11, 12 and 13 participants, respectively. Each focus group had a multidisciplinary composition including different professions as well as family members. Based on the first analysis of these sessions, we concluded that answers were consistent with each other and saturation had been reached, that is, additional focus group sessions were not likely to provide new information. Table 1 shows the participants' characteristics.

**TABLE 1:** Participants' characteristics

Characteristics	Total participants	Family members	Care professionals
	*N* = 49	*N* = 8	*N* = 41
Age [years, mean ± *SD* (min.–max.)]	49 ± 15 (25–76)	71 ± 5 (63–76)	45 ± 12 (25–63)
Sex (% female)	90	63	95
Care institution: Ipse de Bruggen, 's Heeren Loo, Talant, Visio, other (%)	27, 33, 16, 14, 10	38, 13, 13, 13, 25	24, 37, 17, 15, 7
Level of education: mbo, hbo, wo (%)	31, 43, 27	38, 50, 13	29, 41, 29
Role: physician/nurse specialist, DSP, psychologist, allied health care professional, psychologic assistant (%)		N/A	5, 34, 22, 34, 5
Experience working with severe/profound intellectual (and multiple) disabilities [years, mean ± *SD* (min.–max.)]		N/A	15 ± 11 (0.3–43)
Working with severe/profound intellectual (and multiple) disabilities: D, W, M (%)		N/A	61, 37, 2
Working with severe/profound intellectual (and multiple) disabilities + decline/dementia: D, W, M (%)		N/A	37, 44, 20
Family relationship: parent, sibling, legal representative (%)		25, 50, 25	N/A
Characteristics of relative with severe/profound intellectual (and multiple) disabilities			
Age [years, mean ± *SD* (min.–max.)]		56 ± 13 (40–72)	N/A
Presence of Down syndrome (%)		75	N/A
Presence of multiple disabilities (%)		63	N/A
Decline/dementia: yes, no, do not know (%)		75, 13, 13	N/A
Living situation: at home, care institution, combination (%)		13, 63, 25	N/A

*Note*: Percentages (rounded off to the nearest whole number without decimals) are calculated based on the total number of participants per group (column). The group of psychologists is composed of behavioural therapists who studied psychology or special needs education (in Dutch: orthopedagogiek). Occupational therapists, physiotherapists and speech therapists were categorised as allied health care professionals.

Abbreviations: D, daily; DSP, direct support professional/caregiver; hbo, higher vocational education; M, monthly; mbo, intermediate vocational education; N/A, not applicable; W, weekly; wo, higher education.

In the focus groups, participants responded to the three questions. Answers are presented below as descriptions of categories and (sub)themes (question 1/ question 3) or by using a categorisation matrix (question 2).

### 3.1 Question 1: Why is it important to know if an individual with severe/profound intellectual (and multiple) disabilities has dementia?

**FIGURE 1:**
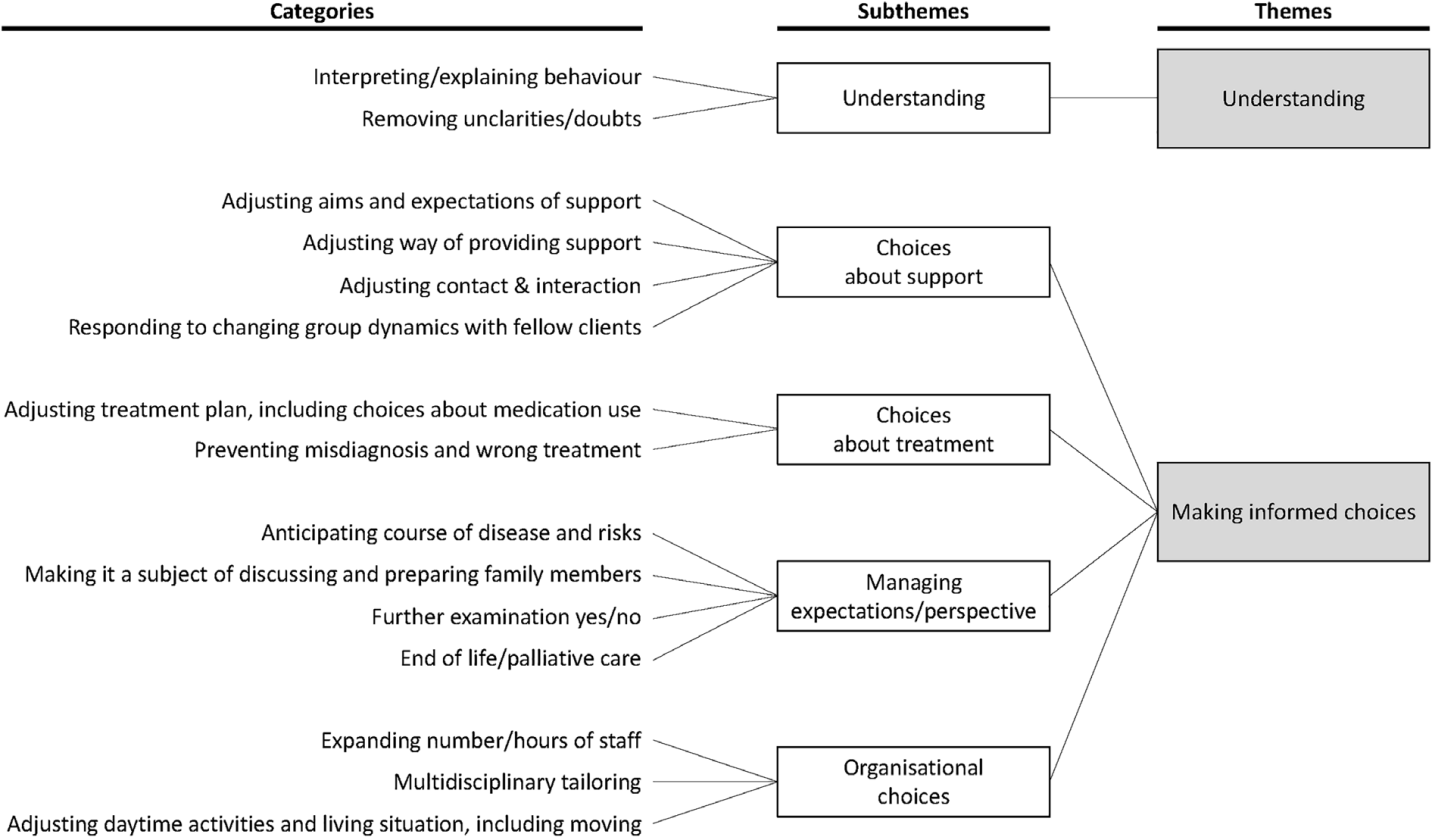
Thematic analysis of the answers to question 1: Why is it important to know if an individual with severe/profound intellectual (and multiple) disabilities has dementia? Figure based on the example in the methodological paper of Elo and Kyngäs (2008)

Thematic analysis revealed two themes (Figure 1): understanding and the ability to make informed choices.

#### 3.1.1 Theme 1.1: Understanding

Participants stated that they want to know whether an individual has dementia in order to be able to explain problematic behaviour.

Psychologist G.: “I am dealing with a man with a severe intellectual disability who is also becoming demented. He tends to display behaviour that is very difficult to understand. Because we know that he is also becoming demented (…) we are better able to comprehend this behaviour, and there is much more sympathy for it”.

#### 3.1.2 Theme 1.2: Making informed choices

When we categorised the codes and divided categories into (sub)themes, it soon became clear that the majority of reasons were more or less related to the ability to make choices. Firstly, it was reported that choices concerned supporting care, for example, adjusting the aims of support, the way support is provided and the way of contact and interaction. Being aware that an individual also has dementia enables participants, for instance, to choose between an activating, development‐oriented way of being supportive and a less development‐oriented approach aimed at monitoring the dementia process and putting emphasis on comfort and maintaining skills. A similar consideration was reported regarding the choice between a behavioural way of being supportive, in which a client is talked to about and persuaded to change their conduct (‘correcting’ the person), and a more monitoring way of being supportive based on the fact that behavioural changes are caused by dementia (‘following’ the person).

Psychologist X.: “[Dementia] means that another approach must be used, in which we do not persuade [clients] to change their behaviour but try to distract them and offer something else”.

DSP C.: “When do you continue to stimulate and when are you taking over? To decide on this, you have to observe someone all day long: what is the client able to do? (…) it is easier to accept that tasks must be taken over from a client who is becoming demented. In that case, you no longer persist in stimulating and assuming that the client is able to do the tasks”.

In addition to choices about support, choices about (medical) treatment were also consistently reported, for example, choices about adjusting a treatment plan and medication use.

DSP E.: “We have a client of whom we are not sure whether she suffers from dementia or depression (…) If she suffers from depression, you may give antidepressants which may revive her. However, if she suffers from dementia, you will need to adjust your actions”.

The population with severe/profound intellectual (and multiple) disabilities is diverse and includes not only people with severe intellectual disabilities who are (somewhat) able to express themselves verbally and move independently, but also people with profound intellectual and multiple disabilities who are not able to talk and are fully dependent on a wheelchair. Some participants raised the question as to whether the label ‘dementia’ would actually change the treatment plan for people with the most severe disabilities. The higher the level of functioning, the more likely it seems that the support and treatment can be adjusted. However, the majority of participants stated that they also wanted to know whether individuals with the most severe disabilities have dementia. In addition to obtaining clarity, it was also mentioned that it is not only about the label but also the preceding thorough diagnostic process. Diagnosing dementia requires a proper (differential) diagnostic procedure. This may also prevent misdiagnosis, which may result in clients receiving the wrong treatment.

The third subtheme concerned management of expectations/perspective. Participants stated that the diagnosis of dementia allows for anticipating the future, for example, anticipating the course of the disease, (timely) entering a conversation with family members to prepare them for what may come and making choices about palliative care and the end of life.

Occupational therapist D.: “The earlier you can discover it, the better. (…) If you know the prognosis, that mental as well as physical [decline] will occur, you can adjust your actions”.

Psychologist E.: “Someone with Alzheimer's disease is, of course, more likely to die sooner. It is uncertain what is going to happen, but I think it may give the family something to hold on to. It is not a pleasant prospect, but it gives you a realistic view of what can happen and the opportunity to inform people about that”.

In view of the prospects, discussions were held about doubts as to whether or not it is useful and necessary to further examine clients, considering the burden and added value of this, for instance.

Father M.: “What can you achieve with all these examinations, how burdensome are they? (…) And how will they explain to our daughter what these tests entail?”

Organisational choices were the final identified subtheme. Participants put forward that recognising dementia may contribute to expanding the number/hours of staff involved and intensifying multidisciplinary collaboration.

Psychologist X.: “You notice that for people with dementia more intensive collaboration is required. It is important that transfer of information takes place more often. Being more in touch with each other, what do we see, what do we hear, which behaviour do we observe, in order to be able to adjust our approach”.

With regard to organisational choices, the dilemma of changing activities during the daytime and/or housing was repeatedly mentioned. Although some participants stated that moving to a suitable house as early as possible enables clients to get used to their new surroundings, others wondered whether you should still introduce changes when dealing with an individual with dementia.

### 3.2 Question 2: Which dementia symptoms in people with severe/profound intellectual (and multiple) disabilities are recognised in daily practice?

The reported dementia symptoms in were coded and subsequently categorised using a matrix (Table 2). Thematic analysis revealed that symptoms were generally observed in the context of nursing (for instance, bathing/showering, toilet use, getting dressed/undressed and external care), eating/drinking, mobility/transfers, communication and leisure activities. In addition, a category called ‘context‐independent’ was created for symptoms of which the context was not sufficiently described or that did not seem to be not specifically related to particular contexts. Cognitive changes were often reported in relation to specific contexts such as nursing, eating/drinking and mobility/transfers. With regard to behavioural changes, symptoms of anxiety were clearly emphasised.

**TABLE 2:** Categorisation matrix structuring dementia symptoms provided by focus group participants

		Contexts					Context‐independent
		Nursing	Eating/drinking	Mobility/transfers	Communication	Leisure activities	
Cognitive changes	Amnesia	No longer understanding what is happening. Cannot remember that you went to the bathroom, where the toilet is, that you have to urinate in the toilet, that you have to go to the toilet, what a garment is for ↓ trained to use the toilet at fixed moments	Inability to make choices. Cannot remember what a spoon/cup is for, what to do with food in your mouth, that you have to eat/drink, that you have to bring food with you when you go to day care, where food has been placed	No longer understanding what is happening. Cannot remember that you were going somewhere, where to go on your bike, where you have put your rollator	Cannot remember what has been said, what a DSP is going to do, what has happened Disguising behaviour: making jokes to cover up what cannot be remembered	Cannot remember what to do with a jigsaw piece, where a new sports venue is, which DSP you need for what	Cannot remember: what is happening around you, where you are in the room ↓ *object permanence* ↑ *letting go of daily structure* ↑ putting objects in another place
	Aphasia			No longer able to tell that they do not know anymore	Sound production changes (tone/note), ↓ talking, ↑ mangling words, ↑ time needed to process what has been said		
	Agnosia		↓ recognition of laid dinner table, spoon, cup, food	↓ *body awareness*, ↓ recognition of rooms, aids		↓ recognition of jigsaw	↓ recognition of DSP, object, ↓ *object permanence*
	Apraxia	No longer able to dry yourself off, put your arm into a sleeve, close your coat, get undressed without help	No longer able to eat/drink properly, use cutlery, pick up/use/put down a cup, continue to drink after stopping for a while, cut bread/stick a fork in the bread, finish plate, leaves food in the mouth(↓ chewing/swallowing)	No longer able to get into the car, use an aid, assume the right sitting position, stand up and take a step, transfer from chamber pot chair to regular chair, move forward Freezing: not able to move anymore		No longer able to do a jigsaw puzzle, exercise	No longer able to perform actions/make movements, such as picking up an object from the floor. ↑ stagnation of actions
	Cognitive other	↓ anticipating steps in getting dressed	↓ awareness of the proper order				↓ overview, ↓ awareness of proper order, ↓ quickness of response,↑ confusion, ↑ disorientation in time/space, colours are perceived differently, alternately bright and not bright
Motor changes	Motor skills	↓ swinging, ↑ stiffness	↓ oral skills ↓ holding spoon	↓ standing up, ↓ turning, ↓ walking, gait changes, ↑ wheelchair use			↓ muscle strength, ↓ motor skills, ↑ cramps
	Balance			↓ balance, ↑ falling			
	Body awareness	↓ *body awareness*		↓ *body awareness*, ↑ slanting			
	Swallowing		↓ swallowing function				
Medical comorbidities		↑ incontinence	↓ weight				↑ incontinence, ↑ pain, ↑ epilepsy, body temperature dysregulation
Behavioural changes^a^	Anxious behaviour	↑ fear of going to bed, fear before/when taking a shower, fear of being touched during nursing (including reliving past sexual trauma during genital care) *Screaming during care*. Holding one's water		↑ fear of transfers (hoist, rising, into wheelchair, reluctant to get out of bed. *Screaming during transfers*. Hesitant to walk/stand (sliding across the floor) or go through the door (fear of crossing thresholds)	↑ monitoring where someone is, ↑ *screaming*, ↑ being frightened, ↑ following DSP, ↑ reassurance seeking		↑ fear of rain, ↑ fear of fellow clients, ↑ *screaming*, ↑ panic, ↑ despair, ↑ *lamenting*, ↑ *crying*, ↑ proximity seeking, ↑ tension, ↑ seeming unhappy, hesitant to let go, no longer feeling safe
	Sleeping problems			↑ prowling about at night, ↑ wandering at night			Disturbance of day–night rhythm, ↑ waking up at night, ↑ sleeping/napping during the day
	Irritable behaviour		↑ getting angry, ↑ throwing away food	↑ running away, ↑ complaining	↑ getting angry, ↑ yelling, ↑ grumbling, ↑ *screaming*, ↑ *groaning*		↑ getting angry, ↑ displeasure, ↑ touchy
	Obstinate behaviour			No longer accepting aids, more/less running away	↑ *groaning*		Rigidity
	Restless and stereotypical behaviour	↑ repeatedly getting undressed			↑ repeating questions		↓ *object permanence*, ↑ restlessness, ↑ speeding up daily programme, increase/decrease in compulsive acts
	Aggressive behaviour						↑ biting, beating, kicking ↑ self‐mutilation
	Apathetic behaviour		↓ focus on eating, drinking	↓ interest in using aids	↑ distancing yourself from the group, ↑ withdrawn into yourself	↓ interest in favourite toy/object, pleasant things, sports	↓ initiative, ↓ interest, ↓ *object permanence*, ↑ masklike face, ↑ *letting go of daily structure*
	Depressive behaviour						↓ enjoying, ↑ emotional, ↑ mood changes, ↑ *crying*, ↑ *lamenting*, ↑ gloominess
	Psychotic behaviour						Visual hallucinations
	Disinhibited behaviour	↓ decorum: pulling up trousers in the hall, getting undressed in front of others	↓ decorum: eating with hands		↑ cuddling, ↑ kissing		Recurrence of problematic behaviour
	Eating/drinking behaviour		↓ appetite, ↓ drinking, ↓ taste sensation, ↑ pica, changes in preferences: ↑ sweet, ↓ hot				

*Note*: ↑, increased; ↓, decreased.

^a^ The categorisation of behavioural and psychological symptoms of dementia is based on the *BPSD‐DS scale* (Dekker et al., 2018, 2021).

In this study, we identified symptoms based on practical experiences instead of neuropsychological assessment. As a result, a number of symptoms could not be uniformly classified because the description was not specific enough or the specific cause could not be ascertained within a focus group session. An example of this is the repeatedly mentioned loss of object permanence, that is, clients who always (have to) carry a certain object with them, for example, a little doll or a cuddly toy, suddenly lose interest in it. This may be due to amnesia (forgetting the object), agnosia (no longer recognising the object), a decrease in compulsory behaviour (the urge to always bring along the object has now subsided) or apathetic behaviour (having lost interest). A few symptoms are, therefore, repeatedly described and italicised in Table 2.

### 3.3 Question 3: What are the training/information needs regarding dementia in people with severe/profound intellectual (and multiple) disabilities?

**FIGURE 2:**
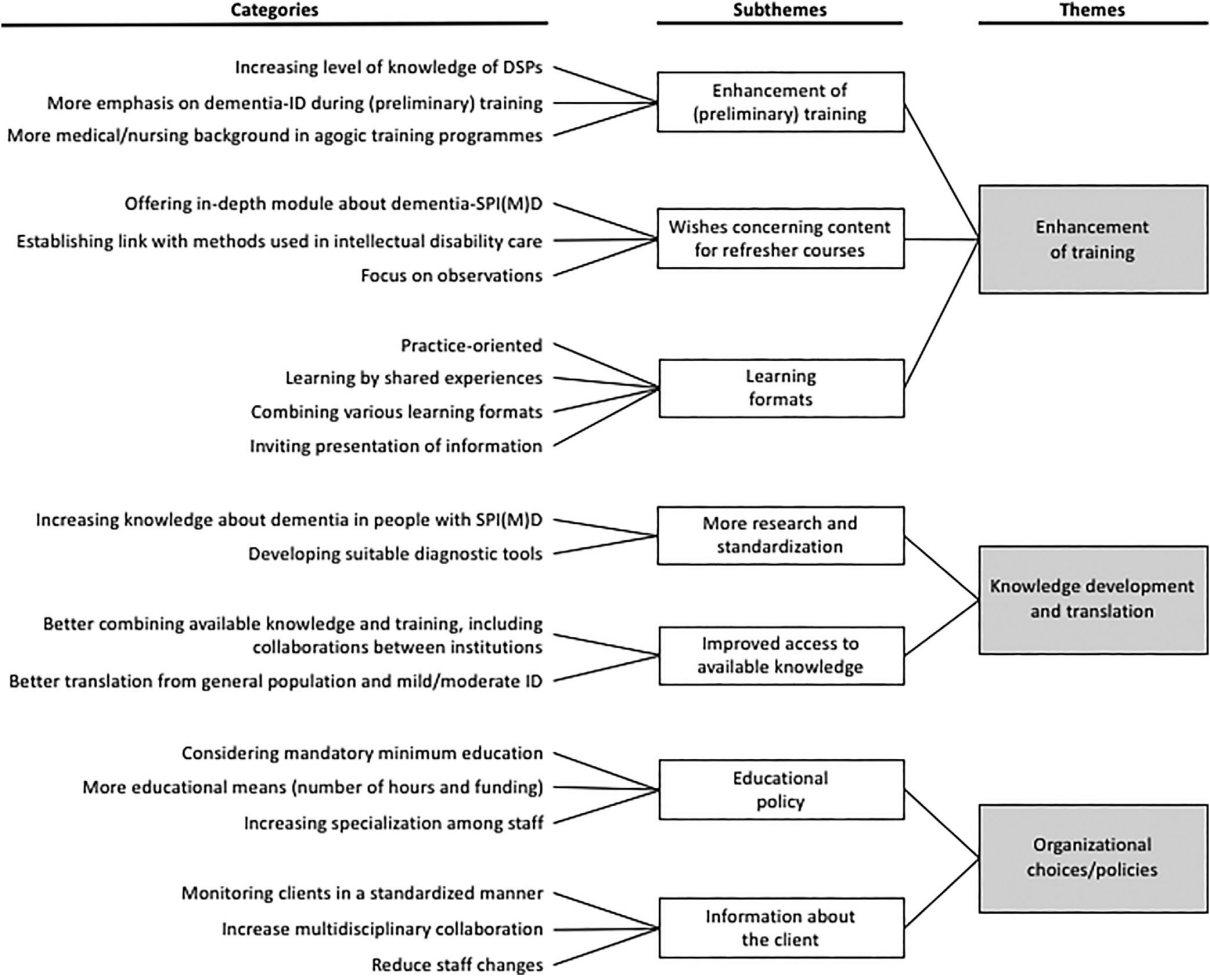
Thematic analysis of the answers to question 3: What are training/information needs regarding dementia in people with severe/profound intellectual (and multiple) disabilities? Figure based on the example in the methodological paper of Elo and Kyngäs (2008)

Thematic analysis revealed three overarching themes: (a) enhancement of training, (b) knowledge development and translation and (c) organisational choices/policies (Figure 2). Participants defined information needs not only in terms of education and knowledge but also in terms of information about the client that they want to have. In addition, participants tended to describe their training needs particularly in terms of problems currently encountered. This was taken into account when phrasing the categories and (sub)themes, so that the question was properly answered.

#### 3.3.1 Theme 3.1: Enhancement of education

Participants stated that the focus of their (preliminary) training had not or hardly been on dementia‐related knowledge. Except for medicine, this goes for intermediate vocational education (mbo) received by most DSPs as well as higher vocational education (hbo) and higher education (wo).

DSP Ch.: “The intermediate vocational education that I received did not focus at all on the ageing client with dementia, let alone dementia and severe intellectual disabilities. It was mainly focused on young people with mild intellectual disabilities”.

Psychologist M.: “Trainees receiving higher vocational education or higher education are completely unaware. When they start their apprenticeship, they think: I am going to administer a standard questionnaire, that is my diagnostic tool. Well, it does not take long to open their eyes”.

It was mentioned that DSPs, who have generally received intermediate vocational education, are expected to be among the first to (early) identify decline. Participants stated that DSPs have usually received agogic training, whereas dementia requires a more medical/nursing background.

DSP C.: “I have colleagues who have only received intermediate vocational education in social care. Although that is very nice, they completely lack experience with dementia (…). It is also preferable to have some individual healthcare or nursing (…) background”.

Physician B: “Medical school focuses a lot on dementia. However, we depend on [information provided by] DSPs. Therefore, they must be fully aware of what they are expected to observe”.

Participants discussed whether specific knowledge about dementia in people with intellectual disabilities, and severe/profound intellectual (and multiple) disabilities in particular, can be (partly) included in (preliminary) training, or whether a specific module about dementia in people with severe/profound intellectual (and multiple) disabilities is more suitable. In addition, participants expressed their needs in terms of training content, including a link with existing methods in intellectual disability care and greater emphasis on observation techniques, such as the repeatedly reported need, when dealing with this population, to pay attention to subtle changes of a client's—often limited—specific functions.

Psychologist R.: “It is necessary to meticulously observe what (…) could previously be accomplished, but not anymore. (…) When dealing with profound intellectual disabilities in particular, these features are key (…) it is advisable to focus on these very critical features. (…) In my opinion, this is a crucial part that is, unfortunately, really lacking”.

With regard to learning formats, participants mentioned that training should be practice‐based (i.e., concrete, easily manageable) and that it should be possible to learn from colleagues' experiences and by doing experience‐based exercises. It was considered desirable to combine various learning formats and to present information in an inviting, for example, visual, manner. It was also stated that e‐learnings do not provide the perfect solution, and it is advisable to combine new learning formats with in‐person meetings.

#### 3.3.2 Theme 3.2: Knowledge development and translation

Participants stated that information about dementia in people with severe/profound intellectual (and multiple) disabilities is lacking. More research is needed into, for instance, the development of (standardised) diagnostic tools suitable for this population, such as dementia questionnaires and the application of video observations to monitor decline.

DSP H.: “I think that the lack of training programmes or additional courses is due to the fact that there is not enough information available about dementia in intellectual disability care. That is the heart of the problem”.

Psychologist E.: “I would like to have some tools, e.g. a questionnaire, (…) instead of the process only taking place in my head (…) I would like to have a standardized tool to help me examine and monitor clients”.

In addition to developing new knowledge, participants stated that they would like to see available knowledge being made more accessible by joining forces more and promoting collaboration between care institutions to prevent them from reinventing the wheel independently from each other. It was also considered desirable that information about dementia in the general population and in people with mild/moderate intellectual disabilities should be translated to severe/profound intellectual (and multiple) disabilities, if possible.

Legal representative F.: “[In regular elderly care] the development of materials regarding dementia is much more advanced. It is not realized that the same approach can be used in people with intellectual disabilities”.

Speech therapist M.: “I think that many organizations set up [training programmes] themselves. However, it may be advisable to combine all these into one new programme”.

#### 3.3.3 Theme 3.3: Organisational choices/policies

Increasing the level of knowledge also depends on choices made by care institutions. Participants stated that time and money should be made available to take courses, and that such courses are often optional and without (many) obligations. It was also mentioned that specialised staff can be of added value to an organisation.

DSP H.: “I regret the fact that going to specialise is not really rewarded. I think that providing a reward might be a way to stimulate staff a little bit more”.

With regard to information needs, it was stated that it is important to systematically monitor clients using standardised methods to improve the transferability of client information. Improvement of multidisciplinary collaboration (also with family members) was emphasised, as well as the importance of reducing staff changes to prevent the loss of knowledge and experience.

Speech therapist A.: “It is always important to document all information gathered about a client. (…) It may be very difficult to make a comparison with the client's previous situation, let's say two years ago, if there are now other DSPs working. (…) Has the situation really deteriorated or got worse?”

Sister M.: “Sometimes, I only talked to the DSP during the annual evaluation meeting. Then I used to think: where is the doctor? Where is the psychologist? Where is the team leader?”

Psychologist G.: “It is very important to adopt a multidisciplinary approach. That it is not only the DSP's responsibility (…) There are so many perspectives that may help to make a good diagnosis”.

## 4 DISCUSSION

In this explorative focus group study on dementia in people with severe/profound intellectual (and multiple) disabilities, we examined the (a) relevance of the diagnosis, (b) symptoms and (c) training/information needs. Thematic analysis revealed that participants want to know whether a person has dementia for a better understanding and to be able to make informed choices. The reported dementia symptoms were categorised using a matrix, in which cognitive changes and behavioural changes were the most prominent. With regard to education, participants expressed their need for enhancement of education, more knowledge development and translation and supportive organisational choices/policies.

The results concerning relevance (question 1) are consistent with Australian research that showed that DSPs often struggle to understand whether behavioural changes are deliberate and people with intellectual disabilities can be called to account for them or whether these are dementia‐related and, therefore, beyond the person's control (Iacono et al., 2014). Knowing whether someone has dementia enables earlier intervention and more appropriate support and resources (Chapman et al., 2018). A few participants wondered whether the label ‘dementia’ would actually change care/treatment for people with the most severe disabilities since they already receive care all their life. Importantly, although dementia cannot (yet) be prevented or cured, non‐pharmacological, psychosocial interventions and (behaviour‐modifying) medication may be used to improve the person's well‐being and quality of life (Bessey & Walaszek, 2019; Keller et al., 2016; MacDonald & Summers, 2020). Without proper diagnosis, treatment might be withheld or the wrong treatment may be provided. Participants disagreed about the use of and need for further testing, for instance in a clinical setting. Consistent with previous research (Chapman et al., 2018), concerns were expressed about the impact on the person.

Not only were choices regarding supportive care and treatment emphasised, but also organisational choices. Whether or not it is advisable to move house was subject of discussion, which was also addressed in scientific literature (Chaput, 2003; Heller et al., 2018; Janicki et al., 2005; Llewellyn, 2011). To enable an individual with dementia to continue to live at a familiar location, adjustments must be made to the house, supportive care and interactions (Chapman et al., 2018; Janicki et al., 2005; Watchman, 2003). According to focus group' participants, a timely diagnosis helps to achieve this.

Although the relevance of the diagnosis was frequently emphasised (question 1), answers to question 3 also showed that the knowledge level needed to identify dementia must be vastly improved. The (timely) identification of signs is almost always the task of DSPs. However, participants stated that there is much room for improvement of DSPs' level of knowledge and expertise, as also found in a review article (Cleary & Doody, 2017). Limited knowledge and the early signs of dementia not being identified may result in delayed dementia diagnosis and subsequent decision‐making delays (Cleary & Doody, 2017), for example, regarding supportive care and treatment. Specific training programmes can improve knowledge, understanding, trust and quality of care (Chapman et al., 2018; Cleary & Doody, 2017). The focus group sessions revealed that this is desirable not only for DSPs but also for professionals from other disciplines that play an important role in the diagnostic process.

The lack of scientific knowledge and dedicated diagnostic tools for dementia in people with severe/profound intellectual (and multiple) disabilities (Elliott‐King et al., 2016; Esbensen et al., 2017; Wissing et al., 2021) on which to base training programmes, makes it more difficult to improve care professionals' knowledge and expertise. As a result, care professionals involved with people with severe/profound intellectual (and multiple) disabilities have to rely on their practice‐based observations, experiences and anecdotal knowledge. Participants emphasised the necessity to develop new knowledge and suitable diagnostic tools. It was frequently stated that knowledge should be made more accessible and closer collaboration is needed between regular elderly care and intellectual disability care, a perspective promoted in literature as well (Heller et al., 2018; Iacono et al., 2014). Participants also emphasised that knowledge development and training cannot take place without making organisational choices and adopting policies focused on dementia in people with intellectual disabilities, such attributing more time to DSPs/carers for individual with dementia (Janicki et al., 2005; Mccarron et al., 2005).

To facilitate the development of new knowledge and diagnostic tools, participants shared their practice‐based symptoms of dementia in people with severe/profound intellectual (and multiple) disabilities. In their daily work, they observe decline across different domains. During these observations, subtle signs are key. The categorisation matrix revealed that cognitive decline and a variety of behavioural changes were observed in particular, which may or may not be associated with specific everyday life situations. Remarkably, participants mentioned a considerable number of cognitive changes, whereas a British study concluded that deterioration in everyday functional skills is more indicative of dementia in people with more severe intellectual disabilities (Jamieson‐Craig et al., 2010). This apparent discrepancy can be explained by the categorisation matrix. By dividing symptoms into deductively ascertained themes (cognitive, behavioural and motor changes; rows) and by linking these to specific contexts inductively obtained from the transcripts (columns), it appears that underlying cognitive changes can be observed in contexts of activities of daily living. Cognitive decline in people with severe/profound intellectual (and multiple) disabilities appears to be mainly apparent during activities of daily living, such as nursing, eating/drinking and mobility/transfers.

### 4.1 Study strengths

To the best of our knowledge, this study is the first to specifically address dementia in people with severe/profound intellectual (and multiple) disabilities. This focus group research is strong because of its solid study design, large number of participants (*N* = 49), multidisciplinary composition and representativeness of intellectual disability care in The Netherlands. Although care professionals in this study were primarily female, this resembles the overrepresentation of women in health care. Indeed, 80% of employees in the Dutch intellectual disability care sector is female (Vereniging Gehandicaptenzorg Nederland, 2019). Within the care institutions, participants were purposefully selected based on their practice‐based experience with decline/dementia in people with severe/profound intellectual (and multiple) disabilities. The structured overview of symptoms is an important first step towards an evidence‐based approach to the diagnosis of dementia in this vulnerable, severely disabled population. This study also provides important information about the relevance of a dementia diagnosis and the training needs of staff and family members. Although training needs were asked in the context of dementia, the (sub)themes emerging from the provided answers (Figure 2) may appear to be applicable to other diseases as well, suggesting that these needs are of essence in good care for people with severe/profound intellectual (and multiple) disabilities in general. For most (sub)themes, the underlying categories specify the needs in the context of dementia.

### 4.2 Study limitations

Considering the multidisciplinary composition, a first limitation was the fact that participation of a physician or nurse specialist could not be achieved in each focus group. Whereas one unspecialized physician and one nurse specialist participated, involvement of specialised intellectual disability physicians would have been desirable.

Secondly, although the focus on people with severe/profound intellectual (and multiple) disabilities was continuously emphasised, care professionals may have referred to some signs of dementia in people with mild/moderate intellectual disabilities (question 2) because they often provide care to people with different levels of functioning. It is also important to mention the considerable heterogenicity of the severe/profound intellectual (and multiple) disabilities population. A number of symptoms, particularly a decline in speech and ability to walk in people with severe intellectual disabilities, were not widely recognised by care professionals who work with clients who are non‐verbal, profoundly disabled and totally dependent on wheelchairs. This underlines the importance of identifying changes within a person by assessing how his/her functioning develops over time. Therefore, a timely baseline measurement of the level of functioning, that is, prior to the occurrence of decline, is essential (Keller et al., 2016).

Thirdly, based on symptoms and contexts mentioned in the transcripts, the categorisation matrix—a simplification of the real situation—was created. However, classification of symptoms was not always straightforward because a detailed description or contextualisation was missing, symptoms appeared to fit into more than one category or a specific cause could not be ascertained within the focus group session. Nevertheless, this is indicative of the struggle faced by participants in daily practice.

A fourth limitation concerned the sudden ending of the audio recording of one of the four focus group sessions approximately 15 min before the session actually ended. However, the fact that some data (question 3) were lost had no impact on saturation.

Finally, in literature there is an ongoing discussion about the necessity to report inter‐rater reliability in qualitative research. Braun and Clarke (2013) argue that reliability is not an appropriate criterion for judging qualitative work, because there is no single true meaning inherent in the data. Instead, to enhance trustworthiness of the analysis, the analysis process and the results should be described in sufficient detail and it is advised to provide authentic citations (Braun & Clarke, 2006; Braun & Clarke, 2013; Elo & Kyngäs, 2008), like in this study.

### 4.3 Future implications

Scientific literature on dementia in people with severe/profound intellectual (and multiple) disabilities has been scarce until now (Wissing et al., 2021), although dementia‐related decline on top of severe/profound intellectual disabilities is very complex. Here, the results clearly emphasise the relevance of research on dementia in this population, show obvious practice‐based needs for more knowledge and suitable diagnostic tools and provide direction for further research. More in‐depth studies of symptoms, for example, medical file analysis and interviews with experienced care professionals to obtain a detailed overview of their practice‐based experiences are needed. Finally, it is important to focus more on dementia in people with severe/profound intellectual (and multiple) disabilities in training programmes. Development of training products related to this topic must be tailored to the wishes and needs in daily practice.

## 5 CONCLUSIONS

This focus group study examined the (a) relevance of the diagnosis, (b) symptoms and (c) training/information needs. It is important to identify dementia (early on) in people with severe/profound intellectual (and multiple) disabilities to be able to make informed choices. To be able to diagnose dementia, a better understanding of dementia symptoms in this population is necessary. This focus group study paves the way for further study of symptoms. In training programmes, dementia in people with severe/profound intellectual (and multiple) disabilities should be incorporated and the provided information should be tailored to practice‐based wishes. People with severe/profound intellectual (and multiple) disabilities are not or hardly able to express the occurrence of deterioration and strongly depend on care professionals. Therefore, improving the knowledge level of these professionals helps to (better) timely identify dementia. As a result, the client's changing wishes and needs can be better responded to by making informed choices.

## CONFLICT OF INTEREST

The authors declare no conflicts of interest.

## AUTHOR CONTRIBUTIONS

Alain D. Dekker: conceptualisation, methodology, resources, investigation, formal analysis, visualization, validation, writing—original draft, writing—review and editing, supervision, project administration, funding acquisition; Maureen B. G. Wissing: formal analysis, visualization, validation, writing—review and editing; Aurora M. Ulgiati: methodology, investigation, formal analysis, visualization, writing—review and editing; Bas Bijl, Gaby van Gool, Marieke R. Groen, Esther S. Grootendorst, and Ina A. van der Wal: resources, investigation, writing—review and editing. Johannes S. M. Hobbelen: writing—review and editing, funding acquisition; Peter P. De Deyn: validation, writing—review and editing, supervision, funding acquisition; Aly Waninge: conceptualization, methodology, resources, investigation, formal analysis, visualization, validation, writing—original draft, writing—review and editing, supervision, project administration, funding acquisition.

## Data Availability

A data management plan has been established for this study. With respect to participants' privacy, data access is restricted and only possible when the applicant meets a set of criteria. A request can be send to the corresponding author. The study consortium will decide whether data can be accessed, among others considering relevance, quality and whether the request meets the conditions of the informed consent. Data are not accessible for commercial purposes.

## References

[jar12912-bib-0001] Alzheimer's Association . (2020). 2020 Alzheimer's disease facts and figures. Alzheimer's and Dementia, 16(3), 391–460. 10.1002/alz.12068 32157811

[jar12912-bib-0002] American Psychiatric Association . (2013). Diagnostic and statistical manual of mental disorders: DSM‐5 (5th ed.). American Psychiatric Association Publishing.

[jar12912-bib-0003] Bessey, L. J. , & Walaszek, A. (2019). Management of behavioral and psychological symptoms of dementia. Current Psychiatry Reports, 21(8), 66. 10.1007/s11920-019-1049-5 31264056

[jar12912-bib-0004] Bittles, A. H. , & Glasson, E. J. (2004). Clinical, social, and ethical implications of changing life expectancy in down syndrome. Developmental Medicine and Child Neurology, 46(4), 282–286. 15077706 10.1017/s0012162204000441

[jar12912-bib-0005] Braun, V. , & Clarke, V. (2006). Using thematic analysis in psychology. Qualitative Research in Psychology, 3(2), 77–101. 10.1191/1478088706qp063oa

[jar12912-bib-0006] Braun, V. , & Clarke, V. (2013). Successful qualitative research: A Practical Guide for Beginners. SAGE Publishing Ltd.

[jar12912-bib-0007] Breen, R. L. (2006). A practical guide to focus‐group research. Journal of Geography in Higher Education, 30(3), 463–475. 10.1080/03098260600927575

[jar12912-bib-0008] Chapman, M. , Lacey, H. , & Jervis, N. (2018). Improving services for people with learning disabilities and dementia: Findings from a service evaluation exploring the perspectives of health and social care professionals. British Journal of Learning Disabilities, 46(1), 33–44. 10.1111/bld.12210

[jar12912-bib-0009] Chaput, J. L. (2003). Adults with down syndrome and Alzheimer' s disease: Comparison of services received in group homes and in special care units. Journal of Gerontological Social Work, 38(1–2), 197–211. 10.1300/J083v38n01_05

[jar12912-bib-0010] Cleary, J. , & Doody, O. (2017). Professional carers' experiences of caring for individuals with intellectual disability and dementia: A review of the literature. Journal of Intellectual Disabilities, 21(1), 68–86. 10.1177/1744629516638245 26976618

[jar12912-bib-0011] Coppus, A. M. W. (2013). People with intellectual disability: What do we know about adulthood and life expectancy? Developmental Disabilities Research Reviews, 18(1), 6–16. 10.1002/ddrr.1123 23949824

[jar12912-bib-0012] Day, K. (1985). Psychiatric disorder in the middle‐aged and elderly mentally handicapped. British Journal of Psychiatry, 147, 660–667. 10.1192/bjp.147.6.660 3938306

[jar12912-bib-0013] Dekker, A. D. , Sacco, S. , Carfi, A. , Benejam, B. , Vermeiren, Y. , Beugelsdijk, G. , Schippers, M. , Hassefras, L. , Eleveld, J. , Grefelman, S. , Fopma, R. , Bomer‐Veenboer, M. , Boti, M. , GDE, O. , Scholten, E. , Tollenaere, M. , Checkley, L. , Strydom, A. , Van Goethem, G. , … De Deyn, P. P. (2018). The behavioral and psychological symptoms of dementia in down syndrome (BPSD‐DS) scale: Comprehensive assessment of psychopathology in down syndrome. Journal of Alzheimer's Disease, 63, 797–820. 10.3233/JAD-170920 PMC5929348 29689719

[jar12912-bib-0014] Dekker, A. D. , Strydom, A. , Coppus, A. M. W. W. , Nizetic, D. , Vermeiren, Y. , Naudé, P. J. W. W. , Van Dam, D. , Potier, M. C. , Fortea, J. , & De Deyn, P. P. (2015). Behavioural and psychological symptoms of dementia in down syndrome: Early indicators of clinical Alzheimer's disease? Cortex, 73, 36–61. 10.1016/j.cortex.2015.07.032 26343344

[jar12912-bib-0015] Dekker, A. D. , Ulgiati, A. M. , Groen, H. , Boxelaar, V. A. , Sacco, S. , Falquero, S. , Carfi, A. , di Paola, A. , Benejam, B. , Valldeneu, S. , Fopma, R. , Oosterik, M. , Hermelink, M. , Beugelsdijk, G. , Schippers, M. , Henstra, H. , Scholten‐Kuiper, M. , Willink‐Vos, J. , de Ruiter, L. , … De Deyn, P. P. (2021). The behavioral and psychological symptoms of dementia in down syndrome scale (BPSD‐DS II): Optimization and further validation. Journal of Alzheimer's Disease, 81(4), 1–23. 10.3233/JAD-201427 PMC8293661 33967040

[jar12912-bib-0016] Duggan, L. , Lewis, M. , & Morgan, J. (1996). Behavioural changes in people with learning disability and dementia: A descriptive study. Journal of Intellectual Disability Research, 40(Pt 4), 311–321. 8884586 10.1046/j.1365-2788.1996.762762.x

[jar12912-bib-0017] Elliott‐King, J. , Shaw, S. , Bandelow, S. , Devshi, R. , Kassam, S. , & Hogervorst, E. (2016). A critical literature review of the effectiveness of various instruments in the diagnosis of dementia in adults with intellectual disabilities. Alzheimer's & Dementia: Diagnosis, Assessment & Disease Monitoring, 4, 126–148. 10.1016/j.dadm.2016.06.002 PMC5061450 27752536

[jar12912-bib-0018] Elo, S. , & Kyngäs, H. (2008). The qualitative content analysis process. Journal of Advanced Nursing, 62(1), 107–115. 10.1111/j.1365-2648.2007.04569.x 18352969

[jar12912-bib-0019] Esbensen, A. J. , Hooper, S. R. , Fidler, D. , Hartley, S. L. , Edgin, J. , d'Ardhuy, X. L. , Capone, G. , Conners, F. A. , Mervis, C. B. , Abbeduto, L. , Rafii, M. S. , Krinsky‐McHale, S. J. , Urv, T. , & Outcome Measures Working Group . (2017). Outcome measures for clinical trials in down syndrome. American Journal on Intellectual and Developmental Disabilities, 122(3), 247–281. 10.1352/1944-7558-122.3.247 28452584 PMC5424621

[jar12912-bib-0020] Evans, E. , Bhardwaj, A. , Brodaty, H. , Sachdev, P. , Draper, B. , & Trollor, J. N. (2013). Dementia in people with intellectual disability: Insights and challenges in epidemiological research with an at‐risk population. International Review of Psychiatry, 25(6), 755–763. 10.3109/09540261.2013.866938 24423228

[jar12912-bib-0021] Evenhuis, H. M. (1990). The natural history of dementia in Down's syndrome. Archives of Neurology, 47(3), 263–267. 10.1001/archneur.1990.00530030029011 2138013

[jar12912-bib-0022] Fletcher, R. J. , Barnhill, J. , McCarthy, J. , & Strydom, A. (2016). From DSM to DM‐ID. Journal of Mental Health Research in Intellectual Disabilities, 9(3), 189–204. 10.1080/19315864.2016.1185324

[jar12912-bib-0023] Heller, T. , Scott, H. M. , Janicki, M. P. , Heller, T. , Esbensen, A. , Fazio, S. , Yoshizaki‐Gibbons, H. , Hartley, D. H. , Janicki, M. P. , Jokinen, N. , Kallmyer, B. , Keller, S. , Magana, S. , Marsack, C. , McCallion, P. , Perkins, E. , Putnam, M. , Qualls, S. , Rader, R. , … Wheeler, B. (2018). Caregiving, intellectual disability, and dementia: Report of the summit workgroup on caregiving and intellectual and developmental disabilities. Alzheimer's and Dementia: Translational Research and Clinical Interventions, 4, 272–282. 10.1016/j.trci.2018.06.002 30090847 PMC6078103

[jar12912-bib-0024] Hon, J. , Huppert, F. A. , Holland, A. J. , & Watson, P. C. (1999). Neuropsychological assessment of older adults with Down's syndrome: An epidemiological study using the Cambridge cognitive examination (CAMCOG). The British Journal of Clinical Psychology/The British Psychological Society, 38(2), 155–165. 10.1348/014466599162719 10389597

[jar12912-bib-0025] Iacono, T. , Bigby, C. , Carling‐Jenkins, R. , & Torr, J. (2014). Taking each day as it comes: Staff experiences of supporting people with down syndrome and Alzheimer's disease in group homes. Journal of Intellectual Disability Research, 58(6), 521–533. 10.1111/jir.12048 23627741

[jar12912-bib-0026] Jamieson‐Craig, R. , Scior, K. , Chan, T. , Fenton, C. , & Strydom, A. (2010). Reliance on carer reports of early symptoms of dementia among adults with intellectual disabilities. Journal of Policy and Practice in Intellectual Disabilities, 7(1), 34–41. 10.1111/j.1741-1130.2010.00245.x

[jar12912-bib-0027] Janicki, M. P. , Dalton, A. J. , Mccallion, P. , Baxley, D. D. , & Zendell, A. (2005). Group home care for adults with intellectual disabilities and Alzheimer's disease. Dementia, 4(3), 361–385. 10.1177/1471301205055028

[jar12912-bib-0028] Keller, S. M. , Janicki, M. P. , & Esralew, L. (2016). Dementia: Screening, evaluation, diagnosis and management. In I. L. Rubin , J. Merrick , D. E. Greydanus , & D. R. Patel (Eds.), Health care for people with intellectual and developmental disabilities across the lifespan (pp. 1449–1463). Springer International. 10.1007/978-3-319-18096-0_116

[jar12912-bib-0029] Llewellyn, P. (2011). The needs of people with learning disabilities who develop dementia: A literature review. Dementia, 10(2), 235–247. 10.1177/1471301211403457

[jar12912-bib-0030] Määttä, T. , Tervo‐Määttä, T. , Taanila, A. , Kaski, M. , & Livanainen, M. (2006). Mental health, behaviour and intellectual abilities of people with down syndrome. Down's Syndrome, Research and Practice: The Journal of the Sarah Duffen Centre, 11(1), 37–43. 10.3104/reports.313 17048808

[jar12912-bib-0031] MacDonald, S. , & Summers, S. J. (2020). Psychosocial interventions for people with intellectual disabilities and dementia: A systematic review. Journal of Applied Research in Intellectual Disabilities, 33, 839–855. 10.1111/jar.12722 32107821

[jar12912-bib-0032] Margallo‐Lana, M. L. , Moore, P. B. , Kay, D. W. K. , Perry, R. H. , Reid, B. E. , Berney, T. P. , & Tyrer, S. P. (2007). Fifteen‐year follow‐up of 92 hospitalized adults with Down's syndrome: Incidence of cognitive decline, its relationship to age and neuropathology. Journal of Intellectual Disability Research, 51(6), 463–477. 10.1111/j.1365-2788.2006.00902.x 17493029

[jar12912-bib-0033] Mccarron, M. , Gill, M. , Mccallion, P. , & Begley, C. (2005). Alzheimer's dementia in persons with Down's syndrome: Predicting time spent on day‐to‐day caregiving. Dementia, 4(4), 521–538. 10.1177/1471301205058305

[jar12912-bib-0034] McKenzie, K. , Metcalfe, D. , & Murray, G. (2018). A review of measures used in the screening, assessment and diagnosis of dementia in people with an intellectual disability. Journal of Applied Research in Intellectual Disabilities, 31, 725–742. 10.1111/jar.12441 29424088

[jar12912-bib-0035] McKhann, G. M. , Knopman, D. S. , Chertkow, H. , Hyman, B. T. , Jack, C. R. , Kawas, C. H. , Klunk, W. E. , Koroshetz, W. J. , Manly, J. J. , Mayeux, R. , Mohs, R. C. , Morris, J. C. , Rossor, M. N. , Scheltens, P. , Carrillo, M. C. , Thies, B. , Weintraub, S. , & Phelps, C. H. (2011). The diagnosis of dementia due to Alzheimer's disease: Recommendations from the National Institute on Aging‐Alzheimer's Association workgroups on diagnostic guidelines for Alzheimer's disease. Alzheimer's &Dementia, 7(3), 263–269. 10.1016/j.jalz.2011.03.005 PMC3312024 21514250

[jar12912-bib-0036] Nakken, H. , & Vlaskamp, C. (2007). A need for a taxonomy for profound intellectual and multiple disabilities. Journal of Policy and Practice in Intellectual Disabilities, 4(2), 83–87. 10.1111/j.1741-1130.2007.00104.x

[jar12912-bib-0037] Rabiee, F. (2004). Focus‐group interview and data analysis. Proceedings of the Nutrition Society, 63(4), 655–660. 10.1079/pns2004399 15831139

[jar12912-bib-0038] Reid, A. H. , & Aungle, P. G. (1974). Dementia in ageing mental defectives: Aclinical psychiatric study. Journal of Mental Deficiency Research, 18(1), 15–23. 10.1111/j.1365-2788.1974.tb01214.x 4278861

[jar12912-bib-0039] Sabbagh, M. , & Edgin, J. (2016). Clinical assessment of cognitive decline in adults with down syndrome. Current Alzheimer Research, 13(1), 30–34. 26391049 10.2174/1567205012666150921095724

[jar12912-bib-0040] Sauna‐aho, O. , Siren, A. , Arvio, M. , Bjelogrlic‐Laakso, N. , Siren, A. , & Arvio, M. (2018). Signs indicating dementia in Down, Williams and Fragile X syndromes. Molecular Genetics & Genomic Medicine, 6(May), 1–6. 10.1002/mgg3.430 PMC6160716 29971948

[jar12912-bib-0041] Sheehan, R. , Sinai, A. , Bass, N. , Blatchford, P. , Bohnen, I. , Bonell, S. , Courtenay, K. , Hassiotis, A. , Markar, T. , McCarthy, J. , Mukherji, K. , Naeem, A. , Paschos, D. , Perez‐Achiaga, N. , Sharma, V. , Thomas, D. , Walker, Z. , & Strydom, A. (2015). Dementia diagnostic criteria in down syndrome. International Journal of Geriatric Psychiatry, 30(8), 857–863. 10.1002/gps.4228 25363568 PMC4678599

[jar12912-bib-0042] Smiley, E. , & Cooper, S.‐A. (2003). Intellectual disabilities, depressive episode, diagnostic criteria and diagnostic criteria for psychiatric disorders for use with adults with learning disabilities/mental retardation (DC‐LD). Journal of Intellectual Disability Research, 47(Suppl 1), 62–71. 14516375 10.1046/j.1365-2788.47.s1.26.x

[jar12912-bib-0043] Strydom, A. , Shooshtari, S. , Lee, L. , Raykar, V. , Torr, J. , Tsiouris, J. , Jokinen, N. , Courtenay, K. , Bass, N. , Sinnema, M. , & Maaskant, M. (2010). Dementia in older adults with intellectual disabilities –Epidemiology, presentation, and diagnosis. Journal of Policy and Practice in Intellectual Disabilities, 7(2), 96–110. 10.1111/j.1741-1130.2010.00253.x

[jar12912-bib-0044] Tong, A. , Sainsbury, P. , & Craig, J. (2018). Consolidated criteria for reporting qualitative research: A 32‐item checklist for interviews and focus groups. International Journal for Quality in Health Care, 19(6), 349–357. 10.1093/intqhc/mzm042 17872937

[jar12912-bib-0045] van Royen, P. , & Peremans, L. (2007). Exploreren met focusgroepgesprekken: de “stem” van de groep onder de loep. In P. L. B. J. Lucassen & T. C. Olde Hartman (Eds.), Kwalitatief onderzoek: Praktische methoden voor de medische praktijk (pp. 53–64). Bohn Stafleu van Loghum.

[jar12912-bib-0046] van Timmeren, E. A. , van der Putten, A. A. J. , van Schrojenstein Lantman‐de Valk, H. M. J. , van der Schans, C. P. , & Waninge, A. (2016). Prevalence of reported physical health problems in people with severe or profound intellectual and motor disabilities: A cross‐sectional study of medical records and care plans. Journal of Intellectual Disability Research, 60(11), 1109–1118. 10.1111/jir.12298 27197564

[jar12912-bib-0047] van Timmeren, E. A. , van der Schans, C. P. , van der Putten, A. A. J. , Krijnen, W. P. , Steenbergen, H. A. , van Schrojenstein Lantman‐de Valk, H. M. J. , & Waninge, A. (2017). Physical health issues in adults with severe or profound intellectual and motor disabilities: A systematic review of cross‐sectional studies. Journal of Intellectual Disability Research, 61(1), 30–49. 10.1111/jir.12296 27228900

[jar12912-bib-0048] Vereniging Gehandicaptenzorg Nederland . (2019). De gehandicaptenzorg in kerngetallen. VGN.

[jar12912-bib-0049] Watchman, K. (2003). Why wait for dementia? Journal of Learning Disabilities, 7(3), 221–230. 10.1177/14690047030073003

[jar12912-bib-0050] Wiseman, F. K. , Al‐Janabi, T. , Hardy, J. , Karmiloff‐Smith, A. , Nizetic, D. , Tybulewicz, V. L. J. , Fisher, E. M. , & Strydom, A. (2015). A genetic cause of Alzheimer disease: Mechanistic insights from down syndrome. Nature Reviews Neuroscience, 16(9), 564–574. 10.1038/nrn3983 26243569 PMC4678594

[jar12912-bib-0053] Wissing, M. B. G. , Ulgiati, A. M. , Hobbelen, J. S. M. , De Deyn, P. P. , Waninge, A. , & Dekker, A. D. (2021). The neglected puzzle of dementia in people with severe/profound intellectual disabilities: A systematic literature review of observable symptoms. Journal of Applied Research in Intellectual Disabilities, 34. 10.1111/jar.12920 PMC9292142 34219327

[jar12912-bib-0051] World Health Organization . (2010). ICD‐10: International statistical classification of diseases and related health problems, (10th Revision). Retrieved fromhttp://apps.who.int/classifications/icd10/browse/2016/en#/F70-F79

[jar12912-bib-0052] Zigman, W. B. , Devenny, D. A. , Krinsky‐McHale, S. J. , Jenkins, E. C. , Urv, T. K. , Wegiel, J. , Schupf, N. , & Silverman, W. P. (2008). Alzheimer's disease in adults with down syndrome. In International review of research in mental retardation (Vol. 36, pp. 103–145). Academic Press, Elsevier Inc. 10.1016/S0074-7750(08)00004-9 19633729 PMC2714652

